# Executive function and food approach behavior in middle childhood

**DOI:** 10.3389/fpsyg.2014.00447

**Published:** 2014-05-19

**Authors:** Karoline Groppe, Birgit Elsner

**Affiliations:** Department of Psychology, Developmental Psychology, University of PotsdamPotsdam, Germany

**Keywords:** hot and cool executive function, eating behavior, food approach, overweight, middle childhood

## Abstract

Executive function (EF) has long been considered to be a unitary, domain-general cognitive ability. However, recent research suggests differentiating “hot” affective and “cool” cognitive aspects of EF. Yet, findings regarding this two-factor construct are still inconsistent. In particular, the development of this factor structure remains unclear and data on school-aged children is lacking. Furthermore, studies linking EF and overweight or obesity suggest that EF contributes to the regulation of eating behavior. So far, however, the links between EF and eating behavior have rarely been investigated in children and non-clinical populations. First, we examined whether EF can be divided into hot and cool factors or whether they actually correspond to a unitary construct in middle childhood. Second, we examined how hot and cool EF are associated with different eating styles that put children at risk of becoming overweight during development. Hot and cool EF were assessed experimentally in a non-clinical population of 1657 elementary-school children (aged 6–11 years). The “food approach” behavior was rated mainly via parent questionnaires. Findings indicate that hot EF is distinguishable from cool EF. However, only cool EF seems to represent a coherent functional entity, whereas hot EF does not seem to be a homogenous construct. This was true for a younger and an older subgroup of children. Furthermore, different EF components were correlated with eating styles, such as responsiveness to food, desire to drink, and restrained eating in girls but not in boys. This shows that lower levels of EF are not only seen in clinical populations of obese patients but are already associated with food approach styles in a normal population of elementary school-aged girls. Although the direction of effect still has to be clarified, results point to the possibility that EF constitutes a risk factor for eating styles contributing to the development of overweight in the long-term.

## Introduction

Self-regulation, which is one of the major achievements in early childhood, is facilitated through a variety of processes which are referred to as executive functions. Executive function (EF) has been found to be strongly (but not exclusively) linked to the prefrontal cortex (PFC; for a meta-analysis see Alvarez and Emory, 2006) and enables the control of thoughts, actions, and emotions (e.g., Zelazo et al., 2008) via a number of related but distinct subfunctions, including shifting, updating, and inhibition (Miyake et al., 2000). EF has long been considered to be a unitary, domain-general cognitive function with its subfunctions working together in a consistent fashion across different situations and content domains (e.g., Zelazo et al., 1997). However, this assumption was partly based on traditional theories emphasizing exclusively one facet of EF measured by relatively abstract, decontextualized problems. More recent research indicates that a different facet of EF is needed when a task involves the regulation of affect and/or motivation (Happaney et al., 2004; Hongwanishkul et al., 2005). Hence, a distinction has been proposed between cognitive “cool” EF, which is activated when solving abstract novel problems, and affective “hot” EF, which is required for problems demanding high affective involvement or flexible appraisals of the affective significance of a stimulus (Zelazo and Müller, 2002).

Evidence for the distinction of hot and cool EF in adults comes from lesion and neuro-imaging studies on diverging functions of different parts of the prefrontal cortex (PFC; Zelazo and Müller, 2002; Happaney et al., 2004). Whereas dorsolateral regions of the PFC (DL-PFC) are associated with cool demands, ventral or medial regions of the PFC (VM–PFC), which are strongly connected to the limbic system, are required for hot regulatory tasks. Furthermore, the distinction is supported by findings that impairments in hot EF can occur in the absence of impairments in cool EF, and vice versa (e.g., Bechara, 2004; Eslinger et al., 2004).

However, to date, empirical findings on hot and cool EF in children remain inconsistent, and further research on its development is needed (Zelazo and Carlson, 2012). There is some indication that changes in cool EF occur earlier than changes in hot EF (e.g., Prencipe et al., 2011), and some studies on preschool-aged children have found that hot and cool EF performance can be described by separate but correlated factors that show different developmental correlates, like academic achievement (e.g., Brock et al., 2009; Willoughby et al., 2011), symptoms of ADHD and behavioral problems, as well as social competence (Sonuga-Barke et al., 2003; Dalen et al., 2004; Smith-Donald et al., 2007). Other studies, however, have found important differences within hot EF tasks, challenging the assumption of a homogeneous hot factor (e.g., Hongwanishkul et al., 2005; Prencipe et al., 2011). Yet other studies found that hot and cool EF do not reflect different factors, but rather belong to a unitary construct in childhood (e.g., Allan and Lonigan, 2011; Wiebe et al., 2011). Some of this inconsistency may come from methodological problems, for instance, most of these studies did not account for the assumption that hot and cool EF are distinct but correlated processes in using either principal-component analyses or varimax rotations in their factor analyses (Willoughby et al., 2011). Moreover, research has to date focused on children younger than 7 years of age, and it might be that the distinction between hot and cool EF emerges later in the course of development, with an increasing functional specialization of neural systems (Johnson, 2011; Zelazo and Carlson, 2012).

To shed further light on the development of EF, the first aim of the present study was to examine whether EF measures can be divided into a hot and a cool factor or whether they correspond to a unitary construct in middle childhood. Because of some evidence for a two-factor structure in younger children (e.g., Brock et al., 2009; Willoughby et al., 2011) and because of the ongoing functional specialization of the neural systems (Johnson, 2011; Zelazo and Carlson, 2012), we expected to find two separate but correlated factors for hot and cool EF. In addition, we tested the factor structure in younger vs. older children of our sample in order to detect age-related differences that may inform about EF development between ages 6 and 11. In particular, we hypothesized that the hot cool distinction might become more evident in older children.

The construct of EF has also received much attention in research on eating disorders and obesity. Overweight and obesity, as well as eating disorders like bulimia and binge-eating disorder, typically involve a dysregulation of eating behavior that points to a prefrontal dysfunction, such as impulsive eating patterns (Spinella and Lyke, 2004). Neurological research supports this interrelation in providing a link between PFC functioning and the control of eating behavior. Imaging studies suggest that the PFC, particularly the VM–PFC, plays a role in different aspects of eating, like affecting the reinforcing value of food, disinhibited eating, hunger, food choice, or weight maintenance (e.g., Tataranni et al., 1999; Appelhans, 2009; Volkow et al., 2009; Cohen et al., 2011; Maayan et al., 2011).

Especially one facet of EF has received further attention in the context of eating, namely the inhibition of dominant responses. Increased impulsivity and reduced inhibitory control are associated with less healthy food choice (e.g., Bryant et al., 2008; Jasinska et al., 2012), eating in response to negative emotional states or external food cues (e.g., Bekker et al., 2004; Elfhag and Morey, 2008) as well as with binge eating (see Fischer et al., 2008; Waxman, 2009 for reviews) and a higher BMI (e.g., Nederkoorn et al., 2006; Batterink et al., 2010).

Furthermore, impairments in various aspects of hot and cool EF have been reported for overweight or obese individuals as compared to normal weight controls, independent of associated medical conditions (see Smith et al., 2011 for a review). For obese children and adolescents (4–18 years), 8 in 9 studies indicate deficits in set shifting, inhibition, working memory, attention, or affective decision-making (Smith et al., 2011). Additionally, there is a link between ADHD and being overweight indicating that EF deficits, as a symptom of ADHD, might favor overeating behaviors (see Cortese et al., 2008; Dempsey et al., 2011, for reviews).

To sum up, results from different research disciplines strongly suggest an association between EF and eating behavior. However, the topic has mostly been examined from a clinical perspective of eating disorders or obesity with the focus on EF deficits in overweight populations compared to controls. The few studies covering EF in relation to eating behavior (and not solely BMI) were limited to examining only inhibition in again mostly clinical populations of adults (e.g., Elfhag and Morey, 2008; Waxman, 2009). To our knowledge, so far, only one study has investigated associations between a broad range of EF and different eating styles in a population sample of adults, and this study reported associations between increased dysexecutive traits and disinhibited eating or greater food cravings (Spinella and Lyke, 2004). This points to a link between EF and eating, even in normal populations, suggesting that eating disorders or obesity represent only the extremes of a normal continuum of eating behavior. Moreover, except for the few studies on obese children (Smith et al., 2011), research on EF and eating or weight issues has almost exclusively focused on adult or adolescent populations. Yet, already children show variation in the extent to which they show food approach behavior, such as food responsiveness, emotional overeating, enjoyment of food, desire to drink, or external eating (Wardle et al., 2001; Sleddens et al., 2008). Illuminating early correlates of such eating behavior that put children at risk for higher weight gain would be of great importance for the prevention of overweight, especially considering the growing prevalence and serious consequences of being overweight and obese (Ogden et al., 2006; Moß et al., 2007).

Therefore, the second aim of the present study was to examine how hot and cool EF are associated with different eating styles that put children at risk of becoming overweight. We expected to find negative associations, i.e., difficulties in self-control, seen in lower levels of hot and cool EF, should co-occur with a higher level of various food approach behaviors in our sample of children in middle childhood. Because other studies have found gender effects for correlates of body weight, such as personality factors (e.g., Brummett et al., 2006; Armon et al., 2013) possible moderations by gender were tested exploratively.

## Methods

### Participants

A total of 1657 children (52.1% girls) aged 6–11 years (*M* = 8.3 years, *SD* = 0.95, *M*d = 8.4 years) and their parents (*N* = 1339) participated in the study. Participants were recruited from 33 elementary schools from the federal state of Brandenburg (German school classes 1–3). Schools were preselected in terms of a representative variety of social backgrounds, as well as urban and rural areas.

Using the criteria of Kromeyer-Hauschild et al. (2001), 81.1% of the children were in the normal BMI range, 6.0% were underweight, 7.7% overweight, and 5.2% obese. This is broadly in line with other prevalence estimates. However, underweight as well as overweight children seemed to be slightly underrepresented (Kurth and Schaffrath Rosario, 2007).

### Material

#### EF measures

***Cool executive functions.*** The attention shifting component of cool EF was measured by the Cognitive Flexibility Task (Roebers et al., 2011; adapted from Zimmermann et al., 2002). Children were told to consecutively feed a plain and a colored fish that appeared simultaneously on the left and right side of a computer screen with randomly changing sides per trial. In order to feed the fish, the child needed to press one of two corresponding keys of a QWERTZ keyboard (the X-key for the left-side fish, the M-key for the right-side fish), remembering which kind of fish had been fed in the previous trial. There were 46 trials (22 switch-trials) separated by a short break that included positive feedback. The interstimuli intervals varied between 300 and 700 ms. The dependent variable for this task was the number of correct responses in the switch-trials (i.e., when the required answer set changed from a right-left to a right-right/left-left reaction, respectively).

The updating component of cool EF (monitoring and updating of working memory representations; Miyake et al., 2000) was assessed by the Digit Span Backwards Task (Petermann and Petermann, 2007). The child heard a sequence of numbers and had to verbally repeat it in reverse order. Each trial consisted of 2 sequences with the same number of digits. The experimenter started with a 2-digit-sequence and passed on to the next trial (one additional digit—except for trial 1 and 2 both consisting of only 2 digits) if at least one of the sequences in a trial had been answered correctly. The dependent variable was the total number of sequences correctly recalled.

The inhibition component of cool EF was measured by the Fruit Stroop Task (Roebers et al., 2011; originally developed by Archibald and Kerns, 1999). Four pages with 25 stimuli each were consecutively presented to the child. Page 1 consisted of colored rectangles (blue, yellow, red, green). Page 2 depicted 4 kinds of fruits and vegetables in appropriate colors (plum = blue, banana = yellow, strawberry = red, lettuce = green). Page 3 presented the same fruits and vegetables but printed in gray. Page 4 again consisted of the same fruits and vegetables, only now they were colored incorrectly. For pages 1 and 2, the children were told to name the color in which items were printed as fast as possible. For pages 3 and 4, children had to name the colors that the fruits/vegetables actually should have (i.e., plum = blue, banana = yellow, etc.). Time (in seconds) needed for naming the colors of all items on each page was measured and an interference score was calculated with higher values indicating more interference: [time p.4 − (time p.1 × time p.3) / (time p.1 + time p.3)] (Archibald and Kerns, 1999).

***Hot executive functions.*** The affective decision-making component of hot EF was measured by an adapted version of the Hungry Donkey Task (Crone and van der Molen, 2004), which is an age-appropriate version of the Iowa Gambling Task, one of the most widely used measures of VM–PFC function (Bechara et al., 1994).

**Figure 1:**
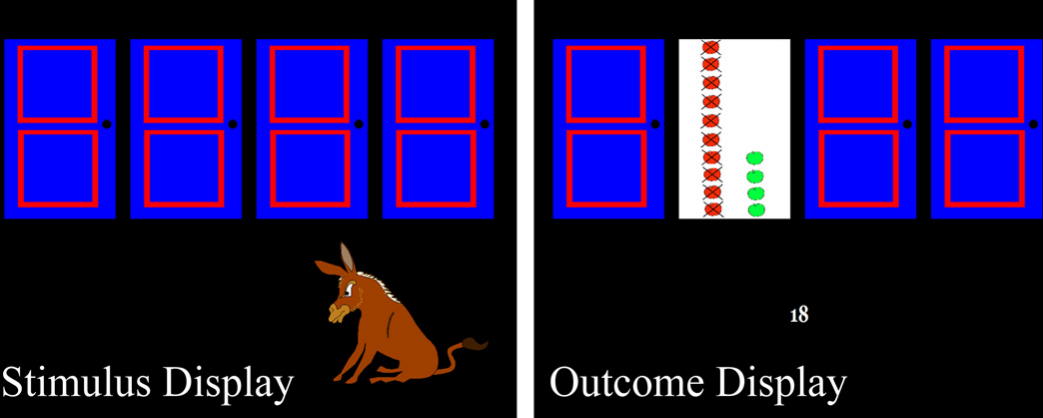
**Stimulus and outcome displays of the Hungry Donkey Task (Crone and van der Molen, 2004)**.

We adapted the task in terms of task duration, instruction, motivational relevance, and complexity, i.e., working memory demands. Four doors (A, B, C, D) were presented side by side on the computer screen (stimulus display; Figure 1). Children were told to assist a hungry donkey to collect as many apples as possible by pressing 1 of 4 keys, opening a corresponding door. Moreover, participants were told that they could win a marble if they collected at least 20 apples (in order to enhance motivational relevance). The S, D, K, and L keys of a QWERTZ keyboard were mapped onto the doors from left to right and the left middle, left index, right index, and right middle fingers were assigned to the keys consecutively. Upon pressing one of the keys an outcome display (Figure 1) was presented at the position of the opened door, showing the number of (green) apples gained and (red and crossed-out) apples lost in the present trial. Furthermore, the overall sum of gained and lost apples across previous trials was displayed as a positive or negative number below the door. The task consisted of 60 trials. Doors A and B (as well as doors C and D) were identical in their underlying win/loss contingencies. Selecting doors A or B resulted in a gain of 4 apples, whereas selecting doors C or D resulted in a gain of only 2 apples. However, doors A and B were disadvantageous in the long run because after selecting doors A or B 10 times, the participant received 40 apples but had also encountered 5 unpredicted losses of 8, 10, 10, 10, or 12 apples, resulting in a net loss of 10 apples. Choosing doors C or D 10 times, in contrast, resulted in a gain of 20 apples with 5 unpredicted losses of 1, 2, 2, 2, or 3 apples, incurring a net gain of 10 apples. The dependent variable was the net-score difference between advantageous and disadvantageous choices [(C+D)–(A+B)] of the last 50 trials (e.g., Crone et al., 2005). The first 10 trials were excluded from the analysis in order to tap decision making under risk, rather than decision making under ambiguity, because win/loss contingencies have probably not yet been experienced during the first trials (Brand et al., 2007)

To measure the delay of gratification component of hot EF children were asked to choose between receiving a smaller reward immediately or a more valuable reward 1 week later (at the second test session; adapted from Wulfert et al., 2002). In 4 trials (1 vs. 2 chocolate drops; 1 vs. 5 chewing candies; 1 vs. 2 bouncing frogs; 1 vs. 3 tattoos), the child always saw the immediate but not the delayed reward. The dependent variable was the number of trials in which the child chose to delay.

In a pretest on 41 children (54% females) aged 8–9 years (*M* = 8.41, *SD* = 0.49) the number of delayed trials showed positive associations in the medium range (*r* = 0.31–0.37, *p* ≤ 0.05) with academic delay of gratification (Academic Delay of Gratification Scale for Children; Zhang et al., 2011), delay of gratification in eating (subscale from the Delaying Gratification Inventory; Hoerger et al., 2011), and impulsivity (German version of Eysenck's I6 Impulsivity Scale; Stadler et al., 2004), indicating good convergent validity. Furthermore, the four trials were highly associated with a longer version of the task (8 items, *r* = 0.88, *p* < 0.001).

#### Food approach behavior and weight assessment

Parents rated the food approach behavior of their children on selected items of 4 scales (3 items each; 5-point-response format: 1 = never, 2 = rarely, 3 = sometimes, 4 = often, 5 = always) of the Children's Eating Behavior Questionnaire (CEBQ; Wardle et al., 2001): Food Responsiveness (e.g., “My child's always asking for food”), Emotional Overeating (e.g., “My child eats more when worried”), Enjoyment of Food (e.g., “My child enjoys eating”), Desire to Drink (e.g., “If given the chance, my child would always be having a drink”), and on the scale External Eating (5 items; e.g., “My child has a desire to eat when s/he watches others eat”) of the Dutch Eating Behavior Questionnaire (DEBQ; Van Strien et al., 1986; 4-point scale: 1 = never, 2 = rarely, 3 = sometimes, 4 = often). Furthermore, the children rated their tendency for Restrained Eating (5 items; e.g., “I try to eat less to avoid weight gain”; DEBQ-C; Franzen and Florin, 1997; Van Strien and Oosterveld, 2008; 4-point scale). Although conceptually, restrained eating is not a food approach behavior, it belongs to a category of eating styles leading to higher weight gain in the long-term (Van Strien and Oosterveld, 2008). Therefore, we subsumed it under the term food approach behavior.

Items of the CEBQ and DEBQ were translated into German and back-translated by a native English speaker. Due to time limits, scales were shortened to 3 (CEBQ), 4 (DEBQ: restrained eating) or 5 (DEBQ: external eating) items with those items being selected that displayed the highest factor loadings. However, a broad content spectrum was intended at the same time. The factorial structure of the two questionnaires remained the same, and internal consistency of scales was acceptable to good (Cronbach's α: 0.71-0.89).

Children's body weight was assessed via calibrated digital scales and height was measured using calibrated ultrasound measurement devices, after shoes, hats and heavy jackets had been removed. A standardized BMI score (BMI-SDS; Kromeyer-Hauschild et al., 2001) was calculated in order to ensure comparability across age and gender.

#### Fluid intelligence

Fluid intelligence was assessed by the Number-Symbol Test of the German version of the Wechsler Intelligence Scale for Children (Petermann and Petermann, 2007).

The child is required to assign symbols to either 5 simple figures (for ages 6–7 years, version A) or to 9 digits (for ages 8–16 years, version B) as quickly as possible. For both versions A and B, the dependent variable is the amount of correct symbols allocated within 120 s (standardized *T*-Values were calculated).

### Procedure

Measures were administered as part of a multifaceted study on intrapersonal developmental risk factors in childhood (February–December 2012). Children completed two 50-min assessments with an interval of about 7 days, conducted by trained and supervised doctoral students or research assistants. Each child was tested individually by one experimenter during the morning hours in a quiet room either at school or at home. Tasks were performed in a counterbalanced order (Blocks of ABCD/BADC). Subsequent analyses, however, revealed no effect of task sequence.

Parents answered the eating behavior questionnaires either online or in printed format. Questionnaires were mostly answered by mothers (71%) or both parents (21%). All participants were guaranteed privacy and children received a cinema voucher as reward upon completion.

All procedures were approved by the Research Ethics Board at the University of Potsdam and by the Ministry of Education, Youth and Sport of the Federal State of Brandenburg. Children and parents were informed about the procedure, materials, and study aims prior to their participation. For each child, informed consent was obtained from a primary caregiver.

### Statistical analyses

Research questions were answered using structural equation modeling (SEM). Models were fit using MPlus Version 7.11 (Muthén and Muthén, 1998–2012). For the first research question we conducted a confirmatory factor analysis (CFA). A one-factor and a two-factor model were fit to the 5 EF tasks. The one-factor model postulated that the tasks can be best described by a unitary higher-level construct. The two-factor model assumed that the tasks can be best conceptualized by a hot and a cool dimension, which are dissociable but correlated. In order to compare the one-factor and the two-factor model on a descriptive level, the model with the lowest Akaike Information Criterion (AIC) was regarded as the best fitting model (Schermelleh-Engel et al., 2003). Furthermore, in order to examine whether the measurement model differs between the younger (<8.4 years) and the older (>8.4 years) half of the sample (median split) we used a χ^2^ difference test to compare a CFA model that estimated factor loadings freely to a CFA model that constrained factor loadings to be equal across groups. The second research question was examined using a SEM in which the 6 eating behavior scales were entered as latent variables and regressed on hot and cool EF, grouping children by gender. Age and fluid intelligence, which are known to be related to EF and eating behavior, were controlled for.

Model fit was evaluated using a combination of absolute (standardized root mean residual, SRMR; root mean squared error of approximation, RMSEA) and comparative (comparative fit index, CFI) fit indices. Model fit was considered good if CFI ≥ 0.97, RMSEA ≤ 0.05 and SRMR ≤ 0.05 (Schermelleh-Engel et al., 2003). We did not rely upon the χ^2^ statistic to evaluate model fit because the value of *p* associated with the χ^2^ statistic is related to sample size and was therefore considered to be overly sensitive to misfits (Schermelleh-Engel et al., 2003). An alpha level of *p* ≤ 0.05 was used for all statistical tests.

The percentage of missing values was ≤1.3 for the child-assessed data (EF, BMI) and ≤ 19.6 for the parent-assessed data (Food Approach). Assuming data to be missing at random we estimated missing values by full information maximum likelihood (FIML) estimation. Results, however, did not differ when analyzing complete cases only.

## Results

### Descriptive statistics and intercorrelations

Bivariate correlations, as well as means and standard deviations, for all of the variables included in structural equation models are summarized in Table 1. On average, children were quite good at solving the attention shifting and the delay of gratification task and they showed medium scores on updating, inhibition, and affective decision-making. The performance on different EF tasks was positively, albeit low to modestly, intercorrelated (Variables 1–5). The 3 cool EF tasks showed low to modest positive correlations with the fluid intelligence measure, whereas the 2 hot EF tasks did not. Furthermore, performance on all EF tasks was positively associated with age. On average, boys showed slightly better performance on the 2 hot EF tasks than did girls, whereas girls outperformed boys in the cool EF inhibition and attention shifting tasks.

**Table 1:** **Descriptive statistics and intercorrelations of assessed variables**.

	**1**	**2**	**3**	**4**	**5**	**6**	**7**	**8**	**9**	**10**	**11**	**12**	**13**	**14**	**15**
1. Attention shifting															
2. Updating	0.33^**^														
3. Inhibition^a^	−0.33^**^	−0.27^**^													
4. Decision making	0.10^**^	0.05^*^	−0.03												
5. Delay of gratification	0.09^**^	0.08^**^	−0.06^*^	0.03											
6. Enjoyment of food	0.01	−0.02	−0.05	−0.02	−0.02										
7. Desire to drink	−0.10^**^	−0.06^*^	0.03	−0.02	0.03	0.05									
8. Food responsiveness	−0.03	−0.03	−0.01	−0.00	−0.03	0.44^**^	0.26^**^								
9. Emotional overeat	0.01	−0.04	0.00	−0.03	0.01	0.10^**^	0.24^**^	0.39^**^							
10. External eating	−0.01	−0.01	0.00	−0.02	0.02	0.41^**^	0.16^**^	0.48^**^	0.27^**^						
11. Restrained eating	−0.06^*^	−0.04	0.03	0.02	0.09	0.08^**^	0.09^**^	0.20^**^	0.08^**^	0.05					
12. BMI-SDS	−0.10^**^	−0.06^*^	0.04	−0.02	−0.01	0.24^**^	0.19^**^	0.44^**^	0.17^**^	0.17^**^	0.30^**^				
13. Male^b^	−0.18^**^	0.02	0.10^**^	0.13^*^	0.09^**^	−0.06	0.03	−0.09^**^	0.03	−0.03	0.02	0.00			
14. Age	0.27^**^	0.22^*^	−0.34^**^	0.06^*^	0.11^*^	0.02	−0.01	0.05	0.01	−0.01	0.01	−0.00	0.04		
15. Fluid intelligence^c^	0.15^**^	0.10^**^	−0.26^**^	−0.00	0.02	0.07^*^	0.02	0.04	0.00	−0.01	−0.03	−0.05^*^	−0.22^**^	−0.07^**^	
Mean	15.6	6.1	24.9	5.5	2.8	3.5	1.9	1.6	1.2	2.9	2.2	0.16	/	8.4	51.4
Standard deviation	4.7	1.6	8.8	11.4	1.2	0.83	0.93	0.89	0.41	0.57	0.84	1	/	0.95	9.2
Min-Max (theoretical)	0–22	0–16	0–89^d^	−60–60	0–4	1–5	1–5	1–5	1–5	1–4	1–4	−4.2–3.6^d^	/	6–11	27–80^d^

N = 1657. Variables 1–5 are indicators of cool (1–3) and hot (4–5) EF; variables 6–11 are facets of food approach behavior.

a Interference measure (negatively polarized);

b Value labels: 1 = male, 0 = female;

c T-Value Number-Symbol-Test;

d Min and/or Max values are theoretically infinite, thus table values are sample-specific.

* p ≤ 0.05;

** p ≤ 0.01.

Children showed low to medium levels of food approach behavior with the highest scores on enjoyment of food. This is broadly in line with results from other studies (Sleddens et al., 2008; Van Strien and Oosterveld, 2008). All food-approach scales were positively correlated with one another and with BMI-SDS, mostly to a medium extent. Girls scored a bit higher than boys on the food responsiveness scale; no other gender differences were apparent.

### Factor structure of EF tasks

#### One-factor (general) vs. two-factor (hot/cool) EF model in the overall sample

**Figure 2:**
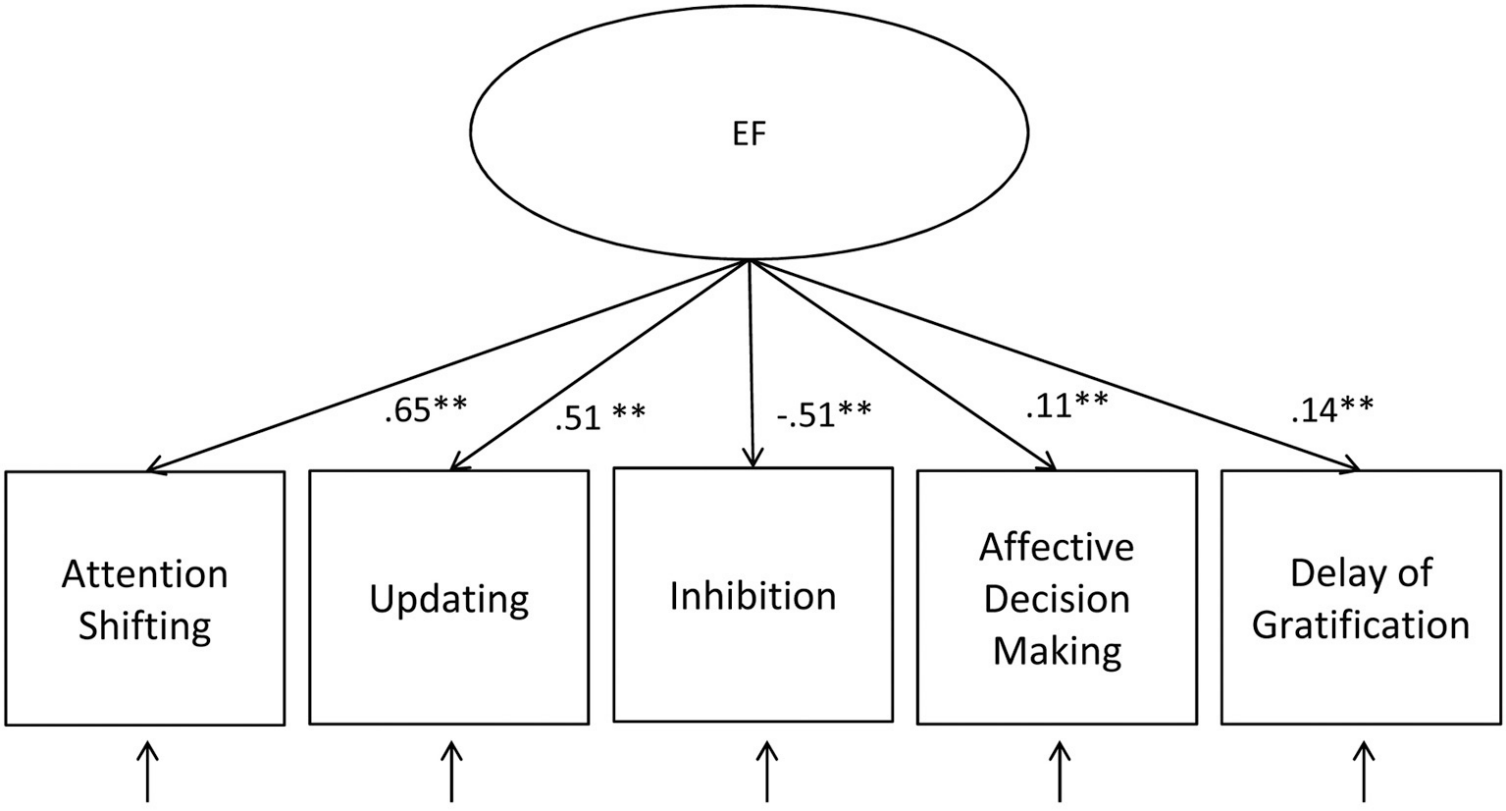
**One-factor CFA model of EF tasks**. ^**^p ≤ 0.01.

The first aim of this study was to examine whether EF measures can be divided into a hot and cool factor or whether they actually correspond to a unitary construct in middle childhood. A one-factor CFA model fitted the data well, χ^2^ (5) = 3.54, *p* = 0.62, *CFI* = 1.00, *RMSEA* = 0.00, *SRMR* = 0.01, AIC = 43,773.59. Standardized parameter estimates are provided in Figure 2. The 3 cool EF tasks showed similar medium-sized factor loadings. However, the standardized factor loadings of the 2 hot EF tasks were significant but very low, falling under a general cutoff value (0.40) for the inclusion into one factor (Stevens, 2001).

**Figure 3:**
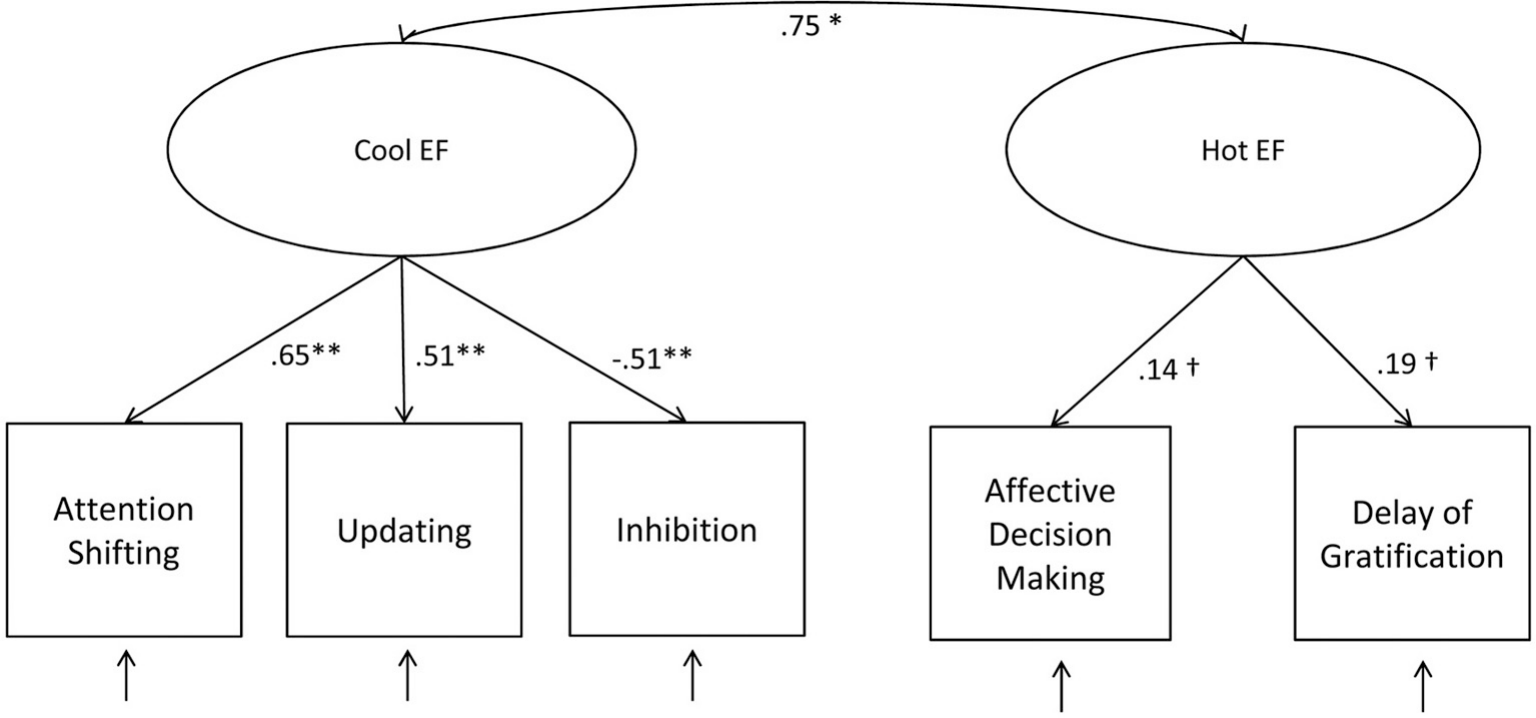
**Two-factor CFA model of EF tasks**. ^†^*p* ≤ 0.10; ^*^p ≤ 0.05; ^**^p ≤ 0.01.

A two-factor CFA model also fitted the data well, χ^2^ (4) = 3.34, *p* = 0.50, *CFI* = 1.00, *RMSEA* = 0.00, *SRMR* = 0.01, *AIC* = 44,775.39. Standardized factor loadings indicated that all 3 cool EF tasks made a nearly equally strong contribution to the cool EF latent variable. However, standardized factor loadings of the 2 hot EF tasks were again very low and only marginally significant indicating that the tasks were not represented well by one underlying hot EF factor. Moreover, there was a high positive correlation between the hot and cool EF latent factors (Figure 3). When comparing models on a descriptive level, the slightly lower AIC value indicated that the one-factor model seemed to be a better tradeoff between model fit and model complexity than the two-factor model.

#### One-factor (general) vs. two-factor (hot/cool) EF model in younger and older children

As a second step, we examined whether the measurement model differed between the younger (<8.4 years) and the older (>8.4 years) half of the sample (median split). Standardized factor loadings for the one-factor and the two-factor model within both age groups are shown in Table 2.

**Table 2:** **Standardized factor loadings for EF tasks on the one- and two-factor model in the younger (<8.4 years) and older (>8.4 years) half of the sample**.

	**1-factor-model**		**2-factor-model**			
	**General-EF factor**		**Hot-EF factor**		**Cool-EF factor**	
	**Younger^b^**	**Older^c^**	**Younger^b^^,^^d^**	**Older^c^**	**Younger^b^^,^^d^**	**Older^c^**
Attention shifting	0.58^**^	0.49^**^			/	0.66^**^
Updating	0.50^**^	0.38^**^			/	0.44^**^
Inhibition^a^	−0.43^**^	−0.61^**^			/	−0.45^**^
Decision making	0.11^*^	0.11^**^	/	0.12		
Delay of gratification	0.12^*^	0.13^**^	/	0.19		

a Interference measure (negatively polarized);

b younger half of the sample;

c older half of the sample;

d two-factor model could not be estimated for the younger half of the sample.

* p ≤ 0.05;

** p ≤ 0.01.

First, the one-factor CFA model was tested in the younger and older age group separately revealing a good fit within both age groups: Younger children: χ^2^ (5) = 3.50, *p* = 0.62, *CFI* = 1.00, *RMSEA* = 0.00, *SRMR* = 0.01; Older children: χ^2^ (5) = 1.63, *p* = 0.90, *CFI* = 1.00, *RMSEA* = 0.00, *SRMR* = 0.01.

Then, a one-factor CFA model that estimated factor loadings freely (A) was tested against a one-factor CFA model that constrained factor loadings to be equal across groups of younger and older children (B) in order to determine whether model fit worsened significantly. Intercepts were constrained to be equal in both models. Both models fitted the data well: Model (A): χ^2^ (14) = 24.15, *p* = 0.04, *CFI* = 0.97, *RMSEA* = 0.03, *SRMR* = 0.03; Model (B): χ^2^ (18) = 27.09, *p* = 0.08, *CFI* = 0.97, *RMSEA* = 0.03, *SRMR* = 0.03. A χ^2^ difference test revealed no significant worsening of fit of the constrained model as compared to the free model, Δχ^2^ (4) = 2.95, *p* = 0.57, suggesting that factor-loadings were equal across groups of younger and older children. In this instance, hot EF loaded very low on the general EF factor in both age groups.

In a second step, a two-factor CFA model was tested separately within both age groups. The two-factor model fitted the data well in the older subgroup, χ^2^ (4) = 1.52, *p* = 0.82, *CFI* = 1.000, *RMSEA* = 0.000, *SRMR* = 0.009, with hot EF factor loadings being again low and not significant. However, it was not possible to estimate the two-factor model in the subgroup of younger children, seemingly due to the absent covariance of hot EF tasks. This suggests that the proposed two-factor model was highly inconsistent with the data, implying that a differentiation of EF into a hot and a cool component does not seem plausible for children aged between 6.0 and 8.4 years in our sample.

### Associations between EF and food approach behavior

The second aim was to examine how hot and cool EF are associated with different eating styles that put children at risk of becoming overweight. We expected that a difficulty in self-control, seen in lower performance in hot and cool EF tasks, would go along with higher occurrence of food approach behavior.

**Figure 4:**
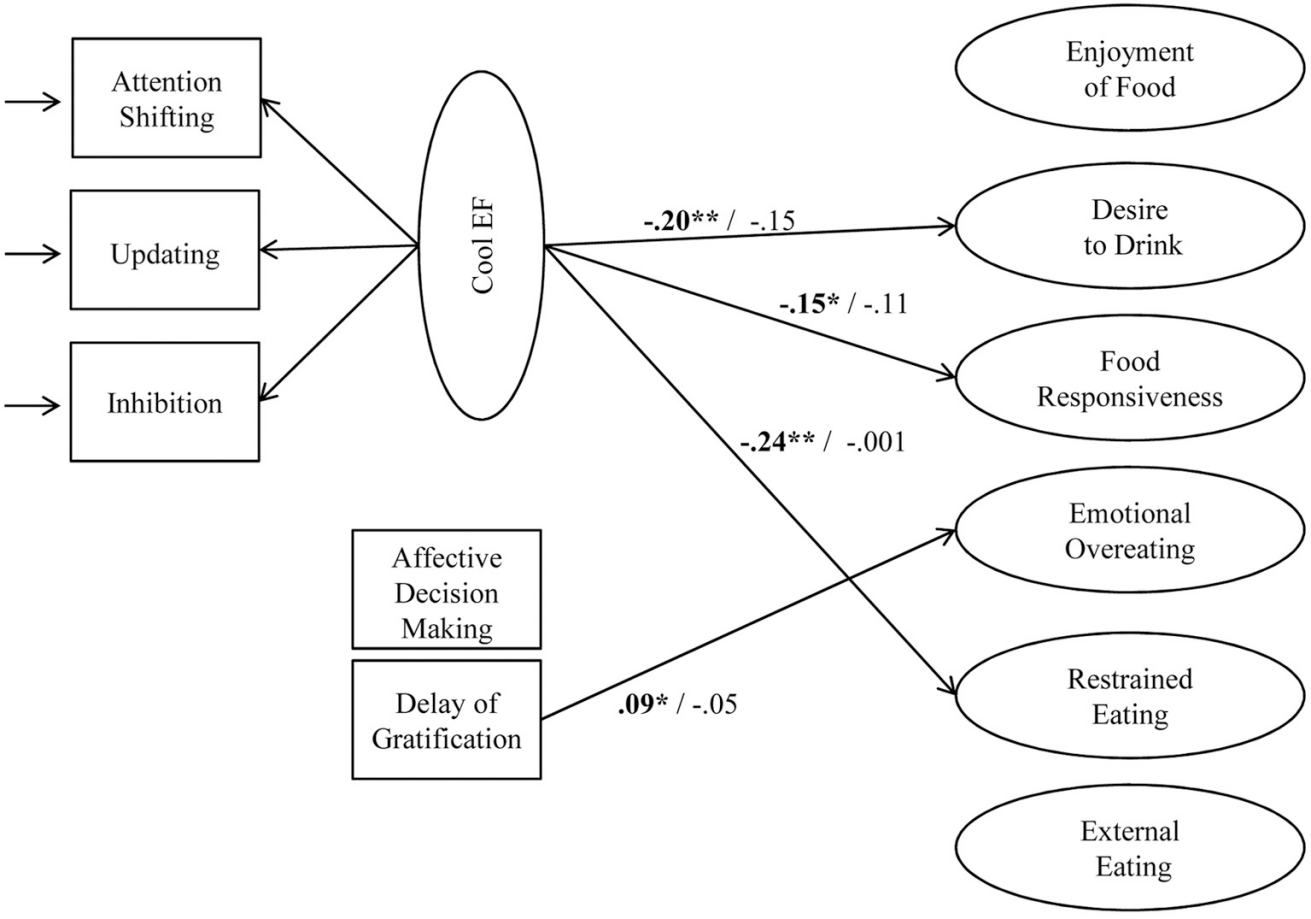
**Associations of the latent cool EF factor and the two hot EF tasks with latent food approach scales**. Results are controlled for age and fluid intelligence. Results of girls are highlighted in bold, results of boys are shown in regular font. ^*^p ≤ 0.05; ^**^p ≤ 0.01.

As the 2 hot EF tasks did neither load well onto one hot EF factor, nor onto the general EF factor, they were further analyzed separately as 2 manifest variables. In contrast, the 3 cool EF tasks were entered as one latent cool EF factor. A SEM was estimated, in which the ratings of children's food approach behavior (6 scales) were regressed on the latent cool EF factor as well as on the 2 manifest hot EF variables, including age (as continuous variable) and fluid intelligence as covariates. Standardized parameter estimates for significant associations (*p* ≤ 0.05) are reported in Figure 4. The SEM fitted the data well, χ^2^ (632) = 1058.31, *p* < 0.01, *CFI* = 0.97, *RMSEA* = 0.03, *SRMR* = 0.04. In girls, cool EF showed relatively small but significant associations with 3 out of 6 eating styles, namely desire to drink, food responsiveness, and restrained eating. Furthermore, the hot EF component delay of gratification was slightly positively related to emotional overeating. However, there were neither significant associations of cool EF and emotional overeating, enjoyment of food, or external eating, nor of the hot EF component affective decision-making and any of the eating styles. In boys, neither hot nor cool EF were significantly associated with any aspect of food approach behavior.

However, using a chi-square difference test to examine differences in regression coefficients between boys and girls revealed a significant moderation by gender only for the association between cool EF and restrained eating, Δχ^2^ (1) = 4.74, *p* = 0.03, and between delay of gratification and emotional overeating, Δχ^2^ (1) = 5.15, *p* = 0.02. Neither the association between cool EF and desire to drink, Δχ^2^ (1) = 0.16, *p* = 0.69, nor between cool EF and food responsiveness, Δχ^2^ (1) = 0.23, *p* = 0.63, differed significantly between boys and girls.

## Discussion

### Structure of hot and cool EF in middle childhood

To date, findings on the structure of hot and cool EF in children have been inconsistent and mostly based on preschool samples. The first aim of this study was to investigate whether performance on EF tasks can be distinguished into correlated hot and cool components (two-factor model) or whether it is better represented by one general EF-factor (one-factor model) in a large sample of children from German school classes 1–3 (aged 6–11 years).

Our data shows that the one-factor as well as the two-factor model fit the data well, with information parameter indices denoting the one-factor EF model to be a better tradeoff between model fit and model complexity. However, standardized factor loadings of the hot EF tasks (affective decision-making, delay of gratification) were very low on both models, falling under a general cutoff value (0.40) for the inclusion into one factor (Stevens, 2001). The cool EF tasks on the other hand showed similar, good factor loadings in both models indicating that the cool components of EF, that is, attention shifting, inhibition, and updating, are highly associated in middle childhood. Comparing subgroups of younger (<8.4 year-olds) and older (>8.4 year-olds) children in our sample, the one-factor model applied to both age groups with equal factor loadings within samples. However, for the younger subgroup only the one-factor-model fit the data well. A two-factor-model could not be estimated due to the missing covariance between the hot EF tasks.

Findings suggest that whereas cool EF seems to be a coherent functional construct in middle childhood, hot EF does not. The two hot EF tasks were neither represented well by the one-factor nor by the two-factor model, which points to the possibility that hot EF is a more complex and heterogeneous construct than originally thought. The minor loadings on the one-factor model indicate that the hot EF tasks did not share a large amount of variance with cool EF, supporting the idea of different underlying mechanisms between hot and cool EF (Zelazo and Müller, 2002). This is further confirmed by differential relations of hot and cool EF to fluid intelligence and gender. Cool but not hot EF tasks were related to a fluid intelligence measure, which has also been proposed previously (e.g., Bar-On et al., 2003; Hongwanishkul et al., 2005). Furthermore, on average, girls outperformed boys in the cool inhibition and attention shifting tasks, whereas boys showed slightly better performance than girls on the two hot EF tasks. The latter is in line with results showing that men outperform women on the Iowa Gambling Task (Reavis and Overman, 2001) and with studies suggesting that VM–PFC develops more rapidly in males than in females (e.g., Clark and Goldman-Rakic, 1989; Overman et al., 1996).

At the same time the minor factor loadings of hot regulatory tasks on a single hot EF factor reflect their missing covariance indicating that hot EF is not a particular homogeneous construct in itself. This confirms negative evidence for a single hot EF factor in younger children, suggesting that the construct of hot EF may need to be further refined (Hongwanishkul et al., 2005; Prencipe et al., 2011). This seems to contradict studies that found substantial correlations within hot EF tasks (e.g., Sonuga-Barke et al., 2003; Smith-Donald et al., 2007; Brock et al., 2009; Willoughby et al., 2011). However, those studies all used variants of delay of gratification tasks to assess hot EF (e.g., snack delay, toy wrap, tongue task) requiring children to wait and inhibit themselves in order to get a reward. In contrast, the two hot EF tasks used in the present study were conceptually less similar, and other studies using variants of those tasks also failed to find evidence for a single hot EF factor. Hongwanishkul et al. (2005) even reported a negative association between the Children's Gambling Task and a delay of gratification task in 3- to 5-year-olds. Similarly, Prencipe et al. (2011) found the Children's Gambling Task not to be associated with a delay discounting task and, consistent with the present results, both tasks loaded only marginally onto a single EF factor in 8- to 11-year-olds. However, in cocaine-dependent adults, the Iowa Gambling Task showed positive relations to delay discounting (Monterosso et al., 2001).

The missing covariance between delay of gratification and affective decision-making can be explained by some fundamental task differences. For instance, both tasks differ considerably in their working memory demands (Hongwanishkul et al., 2005). Whereas the present affective decision-making task required tracking wins and losses across a series of 60 trials, the delay of gratification task involved only 4 independent choices. Furthermore, the two hot EF tasks differed with respect to the time that children had to wait for the rewards and to the certainty with which rewards were obtained. Whereas choice contingencies are clear in the delay of gratification task (one now vs. more 1 week later), they remain purposely unclear in the affective decision-making task (Hongwanishkul et al., 2005).

Moreover, because EF does not develop in a homogenous fashion (e.g., Passler et al., 1985), affective decision-making and delay of gratification, although conceptually related, may evolve at different time points. This would make developmental covariance less likely and is suggested by diverging task difficulties. Whereas the delay of gratification task used in the present study is a rather simple measure of hot EF, the affective decision-making task is relatively complex, probably also placing stronger demands on non-executive skills. This was also reflected by the sample distribution because the delay of gratification task showed some ceiling effects. In contrast, the affective decision-making task proved more difficult to be completed successfully.

Altogether, our results support a distinction between hot and cool facets of EF, but further investigation is needed in order to examine whether hot EF may itself be a heterogeneous construct. This has also been suggested by other authors examining younger and older populations of children (Hongwanishkul et al., 2005; Prencipe et al., 2011). Furthermore, our results on developmental changes in the structure of EF show that whereas performance on all EF tasks was positively associated with age, there was no significant developmental change in the covariance between tasks, disagreeing with the hypothesized idea of a growing differentiation between hot and cool EF for a population of 1st to 3rd graders (Johnson, 2011; Zelazo and Carlson, 2012).

### EF and food approach behavior

The second aim of the present study was to examine whether EF performance is associated with food approach behavior in a population sample of 6- to 11-year-old children. Whereas much is known about EF deficits in clinical populations of the overweight and obese (Smith et al., 2011) there is very little information on how hot and cool EF are associated with eating styles that put children at risk for the development of overweight.

The present study revealed expected negative associations between EF and several food-approach behaviors for girls, but not for boys. After controlling for age and fluid intelligence, in girls lower cool EF went together with higher scores on 3 out of 6 food-approach scales, namely food responsiveness, desire to drink, and restrained eating. No significant associations occurred between cool EF and enjoyment of food, emotional eating, or external eating. Unexpectedly, as for hot EF, performance in the delay of gratification task showed a small *positive* relation to emotional overeating, and the affective decision-making task was not at all associated with food-approach behavior. In boys, neither hot nor cool EF were associated with any of the eating styles. However, the difference in regression coefficients between boys and girls was only significant for the associations between cool EF and restrained eating and between affective decision-making and emotional overeating. All significant regression coefficients were in the low to medium range (Cohen, 1988). However, when interpreting the strength of the associations, the different assessment methods (EF: child experiments vs. food approach: mostly parent ratings) have to be kept in mind (Campbell and Fiske, 1959).

Results show that lower EF cannot only be found in overweight or obese individuals (Smith et al., 2011) but that EF is linearly associated with food-approach styles that are presumed to be a risk factor for the development of overweight in a normal population of children. This is in line with findings that increased dysexecutive traits are associated with disinhibited eating and greater food cravings in a population sample of adults (Spinella and Lyke, 2004). Thus, EF plays a role in eating, even in normal populations of children, suggesting that eating disorders or obesity represent only the extremes of a normal continuum of eating behavior. This is contrary to the assumption of Smith et al. (2011) that negative effects of adiposity on cognition might be only detected in populations who exceed a threshold, i.e., only in the obese.

Although neuroimaging studies suggest that the VM-PFC, which is associated with hot EF, plays a role in the reinforcing value of food, satiety, and the control of eating (Rolls, 2004), we did not find the expected negative associations between hot EF and food approach behavior. However, the facets of hot regulation assessed in our study may not be that relevant for the regulation of eating in normal populations. This might be especially true for the affective decision-making task because its relation to eating on the behavior level is quite subtle.

Furthermore, to date there is only little information on developmental correlates of hot EF. It can be speculated that hot EF will take effect only when severely impaired or over a longer period of time. It might also show its impact on eating only later in development as affective decision-making is believed to develop quite late, with adult levels not being reached until late adolescence (Crone and van der Molen, 2004). This is also reflected in relatively low performance levels in the present sample. Moreover, performance on hot EF tasks may not only result from an inability or cognitive dysfunction but also from unwillingness or a motivational dysfunction (Reynolds and Schiffbauer, 2005; Willoughby et al., 2011), which might bias associations with other variables.

However, we found a low positive association between delay of gratification and emotional overeating. This is surprising and seems to contradict findings showing that obese children have greater difficulty waiting for a larger, delayed reward than children of normal weight (Johnson et al., 1978; Bonato and Boland, 1983). Yet, other authors failed to find such differences between overweight and normal-weight children (Geller et al., 1981; Bourget and White, 1984). However, in a normal child population eating in response to negative emotions might rather be a maladaptive strategy of emotion regulation than an act of impulsivity. Being able to resist a reward requires affect regulation as well, what might explain the small positive association between delay of gratification and emotional overeating. However, this is only speculative and considering the low effect size, this association should not be overrated.

We found associations between cool EF and 3 out of 6 food-approach styles in girls, namely food responsiveness, desire to drink, and restrained eating. Yet, no significant associations occurred between cool EF and enjoyment of food, emotional eating, or external eating. Food responsiveness and desire to drink refer to eating styles that imply a constant need for food or drink. Moreover, restrained eating is often initiated as a response to weight gain (Johnson et al., 2012) probably in order to compensate for lower EF. Thus, those 3 eating styles that were associated with EF might be the more obvious signs of a lack of self-regulation ability as compared to the others.

Associations between EF and food-approach behavior were only found in girls, but not in boys, suggesting that self-regulatory abilities do not play a role in food-approach behavior of elementary school-aged boys. At this age, boys probably self-regulate their own eating behavior less than girls do. This might be due to the facts that in Western cultures the pressure to be thin is much higher for girls and women than for men, and that women face more stringent standards of physical appearance (Friedman and Reichmann, 2002). Women also report more weight stigmatization, starting at lesser degrees of being overweight (e.g., Cossrow et al., 2001) and they suffer more from being overweight than men (Van der Merwe, 2007). Gender differences have also been reported by studies assessing covariates of overweight. For instance, obese girls, but not obese boys, suffer in their ability to focus attention (Mond et al., 2007), suggesting gender-specific associations between obesity and impairments in specific aspects of developmental functioning. Moreover, cross-sectional as well as longitudinal studies found a moderating role of gender in the association between personality factors and body weight (e.g., Brummett et al., 2006; Armon et al., 2013). Positive relations between neuroticism and body weight, and negative relations between conscientiousness and body weight were found to be stronger for women than for men. Likewise, openness was negatively associated with body weight for women, but not for men. Although the present findings did not reveal any relations between EF and food-approach behavior in boys, these might just occur at a later age, as soon as pubertal development makes dealing with body-weight issues and the resulting conscious regulation of eating more relevant for boys.

One limitation of the present study is that children's performance in hot and cool EF was measured by only 5 tasks. Future studies would benefit from the inclusion of a greater number of indicators, especially for hot EF in order to further examine possible differences within hot EF. Moreover, although the hot EF tasks were mainly not associated with food approach behavior in our sample, this does not imply that hot EF is less important than cool EF for the regulation of eating. Probably, applying ecologically more relevant tasks could have helped to show associations of hot EF with eating behavior.

### Conclusion

The present study examined the structure of hot and cool EF and its relation to food approach behavior in a representative sample of elementary-school children from school class 1–3. Results showed that cool EF seems to be a reasonable coherent functional construct in middle childhood. However, further clarification is required regarding the construct of hot EF. Nevertheless, hot and cool EF do not seem to share exactly the same underlying mechanisms, and their distinction is supported by differential relations to fluid intelligence and food-approach behavior, as well as by gender differences in task performance. Therefore, as has been noted by other authors (e.g., Hongwanishkul et al., 2005), it needs to be further examined to what extent hot EF—although distinct from cool EF—might not be a homogeneous construct itself.

Furthermore, the study provides first evidence that not only obesity is associated with impaired EF, but that linear associations between hot and cool EF and the occurrence of food approach behaviors occur in a normal population of elementary school-aged girls. This extends findings on relationships of prefrontal neural systems and eating from clinical populations, e.g., patients showing neurological or eating disorders (e.g., Dempsey et al., 2011; Smith et al., 2011), into the normal population. Considering these results, it seems plausible to assume that EF constitutes a risk factor for eating styles that contribute to the development of overweight. However, results of the present study rely on cross-sectional data. Longitudinal designs examining relations between earlier EF and later eating behavior are needed to shed light on the important question of whether EF is a risk factor for the development of obesity or whether in turn the type of diet is responsible for cognitive deficits (Smith et al., 2011).

Today's oversupply of palatable high-caloric food is known to play an important role in promoting obesity (Hill and Peters, 1998) but not all individuals exposed to this environment become overweight or obese. Determining modifiable risk factors of obesity is of particular importance given that obesity is currently considered one of the most increasingly important health issues (WHO, 2006; Moß et al., 2007). As there is evidence that EF capacity can be improved (e.g., Klingberg et al., 2005; Diamond and Barnett, 2007) and that EF improvement helps patients suffering from eating disorders (Tchanturia et al., 2007; Genders et al., 2008), the training of EF appears to be a promising tool for the prevention of overweight and obesity in children. Thus, examining the exact role of EF for the development of obesity seems to be an important topic for future research.

## Author contributions

All authors have contributed considerably to the conception and design of the work including the formulation of hypotheses. Karoline Groppe has primarily analyzed and interpreted the data, whereas Birgit Elsner has formulated the problem and revised the work. Both authors have agreed to be accountable for all aspects of the work and to submit the manuscript in this form.

### Conflict of interest statement

The authors declare that the research was conducted in the absence of any commercial or financial relationships that could be construed as a potential conflict of interest.
